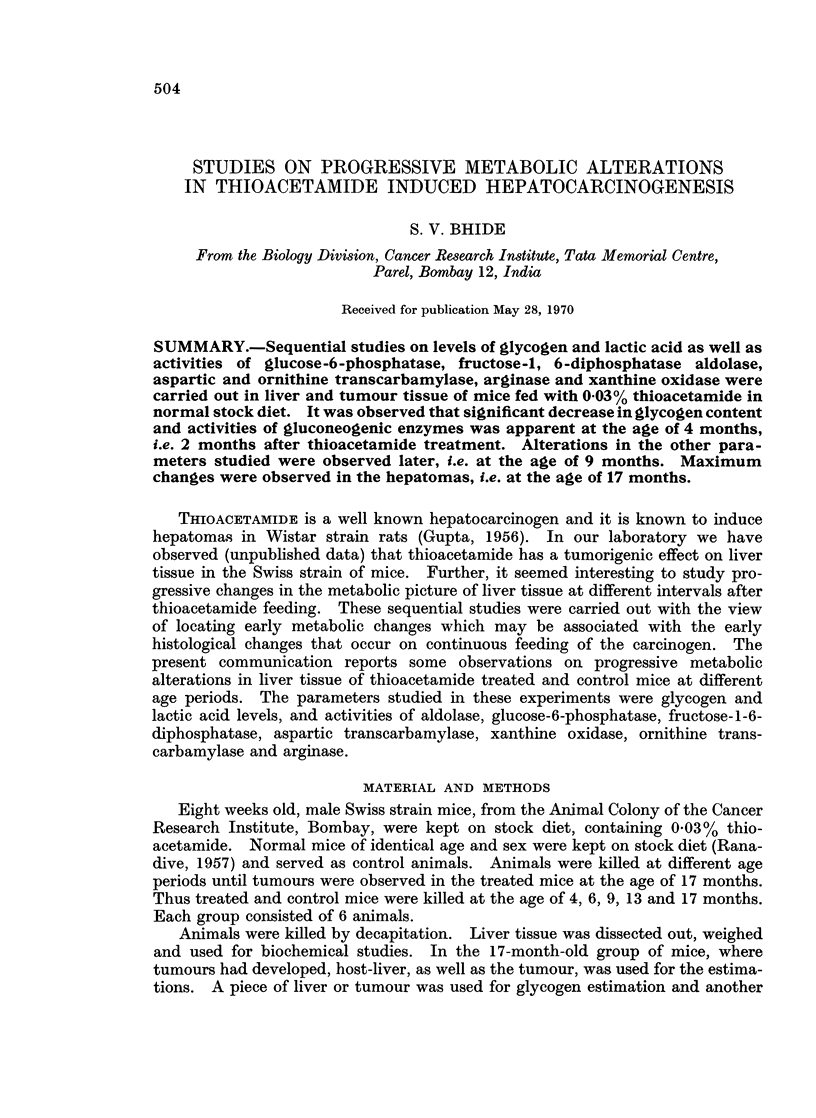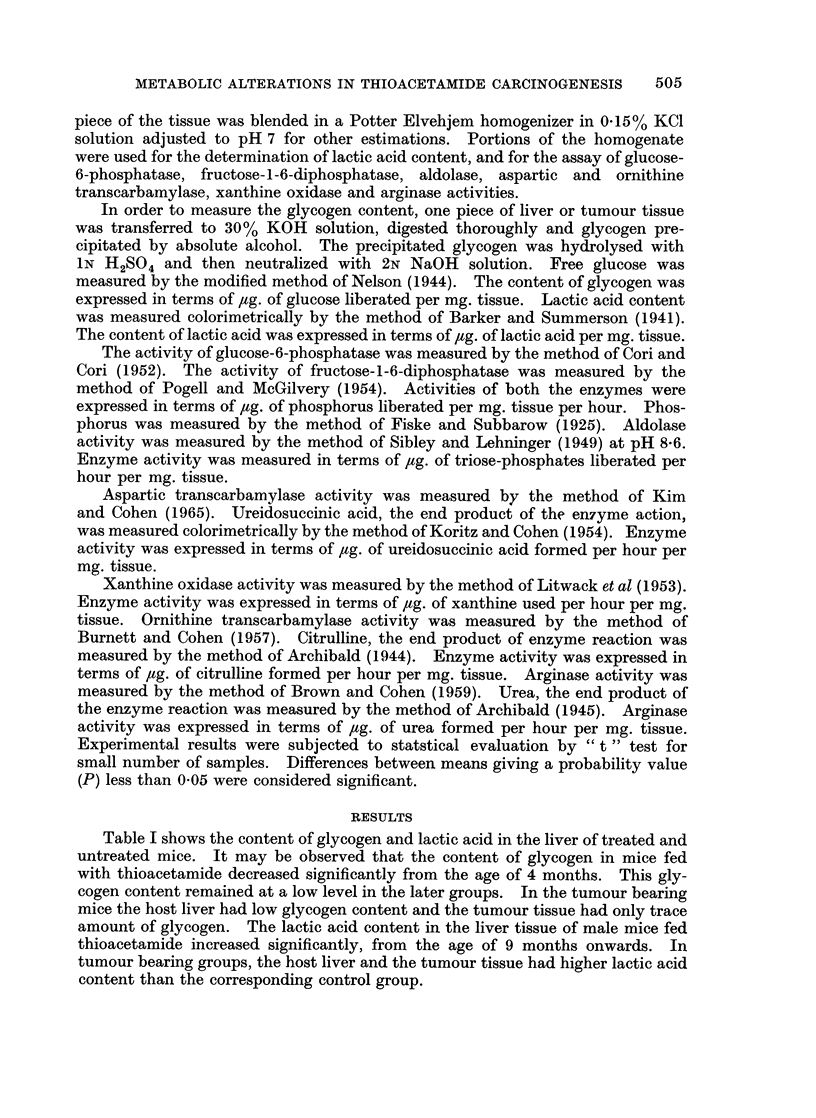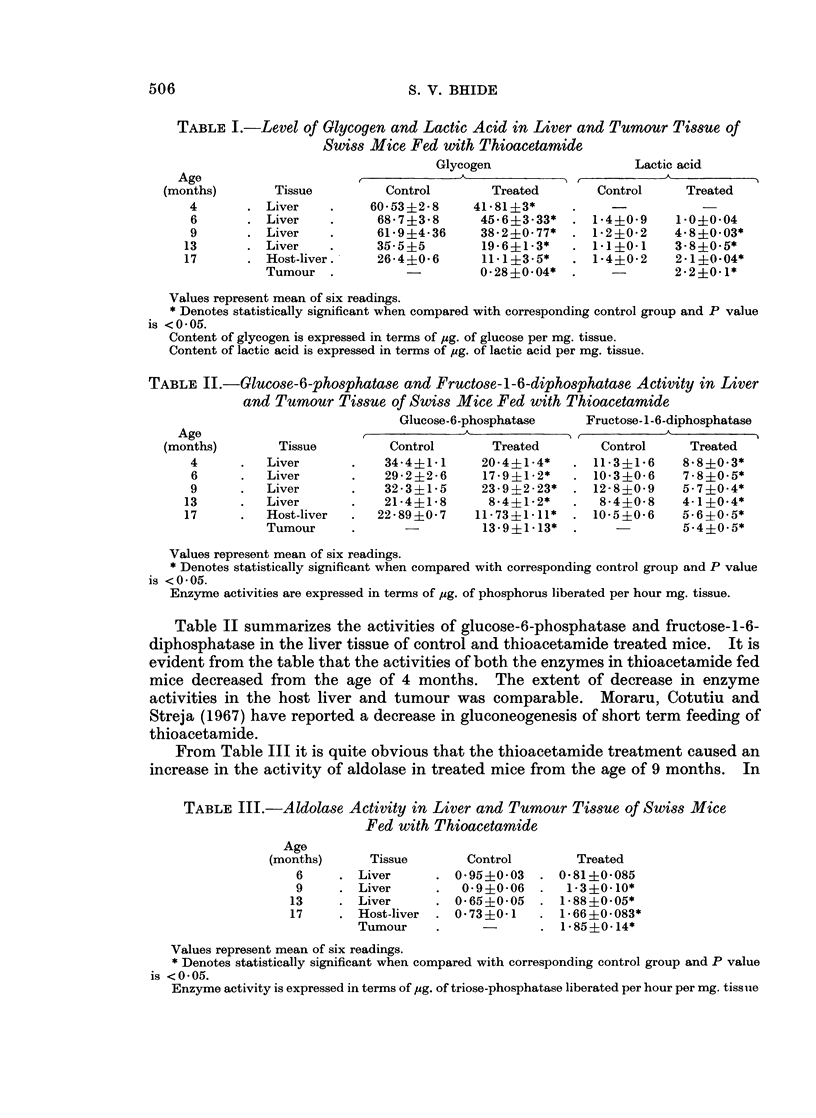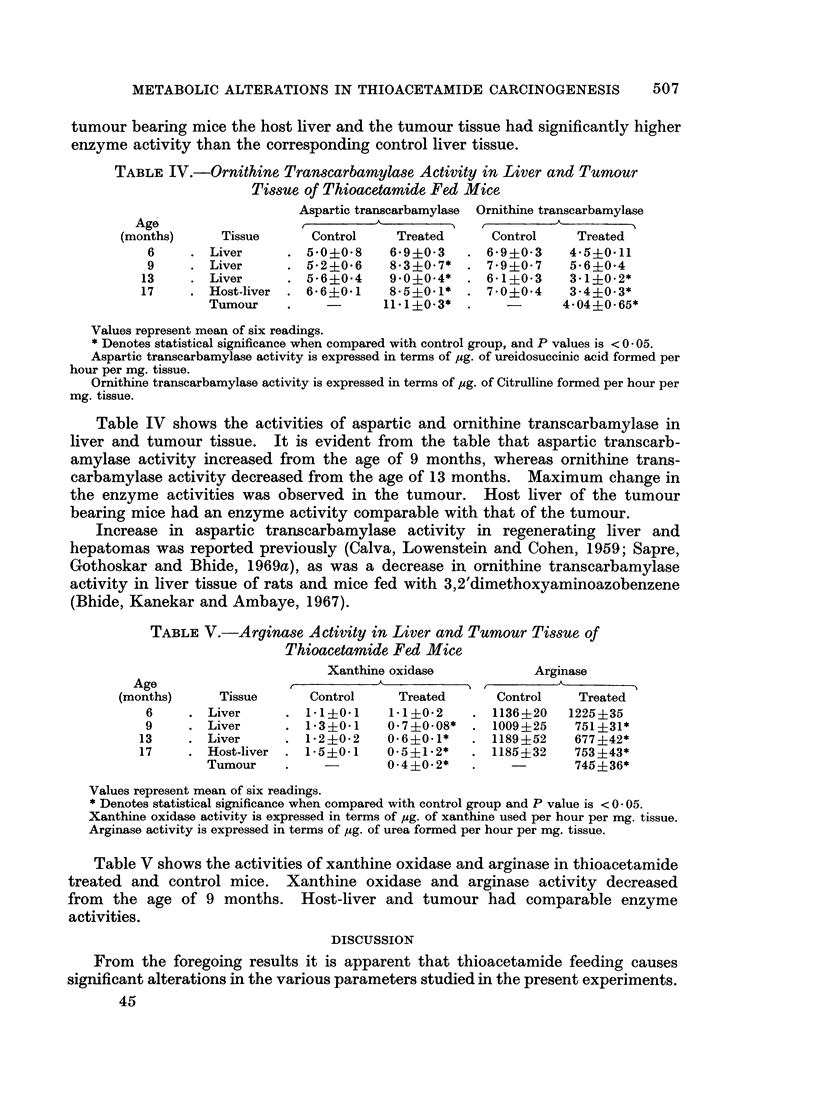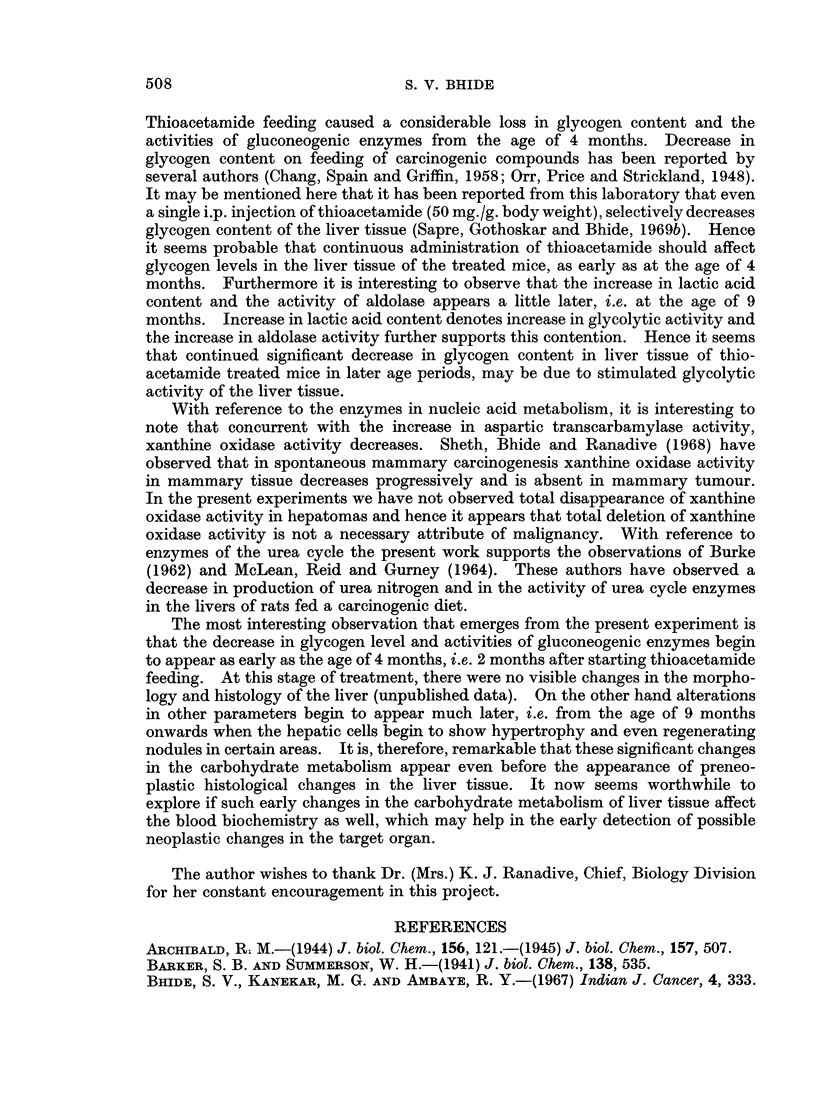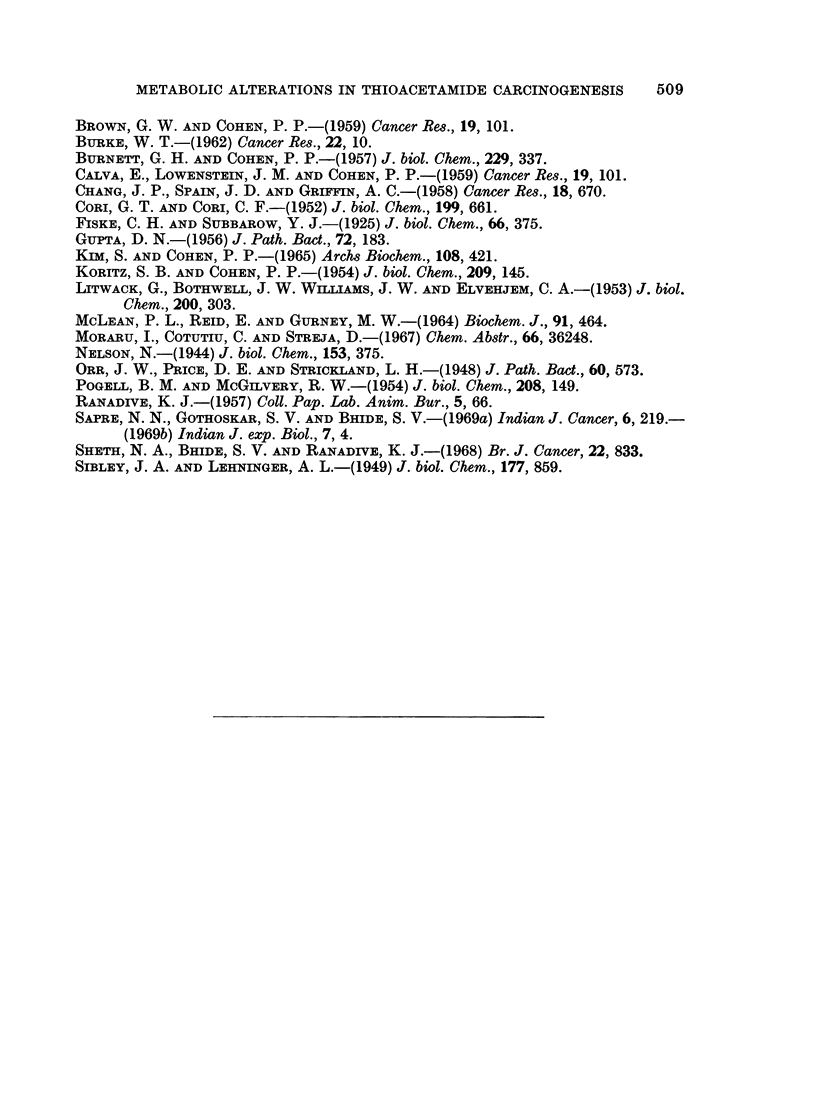# Studies on Progressive Metabolic Alterations in Thioacetamide Induced Hepatocarcinogenesis

**DOI:** 10.1038/bjc.1970.61

**Published:** 1970-09

**Authors:** S. V. Bhide

## Abstract

Sequential studies on levels of glycogen and lactic acid as well as activities of glucose-6-phosphatase, fructose-1, 6-diphosphatase aldolase, aspartic and ornithine transcarbamylase, arginase and xanthine oxidase were carried out in liver and tumour tissue of mice fed with 0.03% thioacetamide in normal stock diet. It was observed that significant decrease in glycogen content and activities of gluconeogenic enzymes was apparent at the age of 4 months, *i.e.* 2 months after thioacetamide treatment. Alterations in the other parameters studied were observed later, *i.e.* at the age of 9 months. Maximum changes were observed in the hepatomas, *i.e.* at the age of 17 months.


### STUDIES ON PROGRESSIVE METABOLIC ALTERATIONS IN THIOACETAMIDE INDUCED HEPATOCARCINOGENESIS

S. V. BHIDE

*From the Biology Division, Cancer Research Institute, Tata Memorial Centre, Parel, Bombay* 12, *India*

Received for publication May 28, 1970

**SUMMARY.—Sequential studies on levels of glycogen and lactic acid as well as activities of glucose-6-phosphatase, fructose-1, 6-diphosphatase aldolase, aspartic and ornithine transcarbamylase, arginase and xanthine oxidase were carried out in liver and tumour tissue of mice fed with 0·03% thioacetamide in normal stock diet. It was observed that significant decrease in glycogen content and activities of gluconeogenic enzymes was apparent at the age of 4 months, *i.e.* 2 months after thioacetamide treatment. Alterations in the other parameters studied were observed later, *i.e.* at the age of 9 months. Maximum changes were observed in the hepatomas, *i.e.* at the age of 17 months.**

THIOACETAMIDE is a well known hepatocarcinogen and it is known to induce hepatomas in Wistar strain rats (Gupta, 1956). In our laboratory we have observed (unpublished data) that thioacetamide has a tumorigenic effect on liver tissue in the Swiss strain of mice. Further, it seemed interesting to study progressive changes in the metabolic picture of liver tissue at different intervals after thioacetamide feeding. These sequential studies were carried out with the view of locating early metabolic changes which may be associated with the early histological changes that occur on continuous feeding of the carcinogen. The present communication reports some observations on progressive metabolic alterations in liver tissue of thioacetamide treated and control mice at different age periods. The parameters studied in these experiments were glycogen and lactic acid levels, and activities of aldolase, glucose-6-phosphatase, fructose-1-6-diphosphatase, aspartic transcarbamylase, xanthine oxidase, ornithine transcarbamylase and arginase.

### MATERIAL AND METHODS

Eight weeks old, male Swiss strain mice, from the Animal Colony of the Cancer Research Institute, Bombay, were kept on stock diet, containing 0·03% thioacetamide. Normal mice of identical age and sex were kept on stock diet (Ranadive, 1957) and served as control animals. Animals were killed at different age periods until tumours were observed in the treated mice at the age of 17 months. Thus treated and control mice were killed at the age of 4, 6, 9, 13 and 17 months. Each group consisted of 6 animals.

Animals were killed by decapitation. Liver tissue was dissected out, weighed and used for biochemical studies. In the 17-month-old group of mice, where tumours had developed, host-liver, as well as the tumour, was used for the estimations. A piece of liver or tumour was used for glycogen estimation and another piece of the tissue was blended in a Potter Elvehjem homogenizer in 0·15% KCl solution adjusted to pH 7 for other estimations. Portions of the homogenate were used for the determination of lactic acid content, and for the assay of glucose-6-phosphatase, fructose-1-6-diphosphatase, aldolase, aspartic and ornithine transcarbamylase, xanthine oxidase and arginase activities.

In order to measure the glycogen content, one piece of liver or tumour tissue was transferred to 30% KOH solution, digested thoroughly and glycogen precipitated by absolute alcohol. The precipitated glycogen was hydrolysed with 1N $H_2SO_4$ and then neutralized with 2N NaOH solution. Free glucose was measured by the modified method of Nelson (1944). The content of glycogen was expressed in terms of $\mu$g. of glucose liberated per mg. tissue. Lactic acid content was measured colorimetrically by the method of Barker and Summerson (1941). The content of lactic acid was expressed in terms of $\mu$g. of lactic acid per mg. tissue.

The activity of glucose-6-phosphatase was measured by the method of Cori and Cori (1952). The activity of fructose-1-6-diphosphatase was measured by the method of Pogell and McGilvery (1954). Activities of both the enzymes were expressed in terms of $\mu$g. of phosphorus liberated per mg. tissue per hour. Phosphorus was measured by the method of Fiske and Subbarow (1925). Aldolase activity was measured by the method of Sibley and Lehninger (1949) at pH 8·6. Enzyme activity was measured in terms of $\mu$g. of triose-phosphates liberated per hour per mg. tissue.

Aspartic transcarbamylase activity was measured by the method of Kim and Cohen (1965). Ureidosuccinic acid, the end product of the enzyme action, was measured colorimetrically by the method of Koritz and Cohen (1954). Enzyme activity was expressed in terms of $\mu$g. of ureidosuccinic acid formed per hour per mg. tissue.

Xanthine oxidase activity was measured by the method of Litwack *et al* (1953). Enzyme activity was expressed in terms of $\mu$g. of xanthine used per hour per mg. tissue. Ornithine transcarbamylase activity was measured by the method of Burnett and Cohen (1957). Citrulline, the end product of enzyme reaction was measured by the method of Archibald (1944). Enzyme activity was expressed in terms of $\mu$g. of citrulline formed per hour per mg. tissue. Arginase activity was measured by the method of Brown and Cohen (1959). Urea, the end product of the enzyme reaction was measured by the method of Archibald (1945). Arginase activity was expressed in terms of $\mu$g. of urea formed per hour per mg. tissue. Experimental results were subjected to statstical evaluation by "t" test for small number of samples. Differences between means giving a probability value ($P$) less than 0·05 were considered significant.

### RESULTS

Table I shows the content of glycogen and lactic acid in the liver of treated and untreated mice. It may be observed that the content of glycogen in mice fed with thioacetamide decreased significantly from the age of 4 months. This glycogen content remained at a low level in the later groups. In the tumour bearing mice the host liver had low glycogen content and the tumour tissue had only trace amount of glycogen. The lactic acid content in the liver tissue of male mice fed thioacetamide increased significantly, from the age of 9 months onwards. In tumour bearing groups, the host liver and the tumour tissue had higher lactic acid content than the corresponding control group.

TABLE I.—*Level of Glycogen and Lactic Acid in Liver and Tumour Tissue of Swiss Mice Fed with Thioacetamide*

| Age (months) | Tissue | Glycogen Control | Glycogen Treated | Lactic acid Control | Lactic acid Treated |
|---|---|---|---|---|---|
| 4 | Liver | 60·53±2·8 | 41·81±3* | — | — |
| 6 | Liver | 68·7±3·8 | 45·6±3·33* | 1·4±0·9 | 1·0±0·04 |
| 9 | Liver | 61·9±4·36 | 38·2±0·77* | 1·2±0·2 | 4·8±0·03* |
| 13 | Liver | 35·5±5 | 19·6±1·3* | 1·1±0·1 | 3·8±0·5* |
| 17 | Host-liver | 26·4±0·6 | 11·1±3·5* | 1·4±0·2 | 2·1±0·04* |
| | Tumour | — | 0·28±0·04* | — | 2·2±0·1* |

Values represent mean of six readings.
* Denotes statistically significant when compared with corresponding control group and $P$ value is $<0·05$.
Content of glycogen is expressed in terms of $\mu$g. of glucose per mg. tissue.
Content of lactic acid is expressed in terms of $\mu$g. of lactic acid per mg. tissue.

TABLE II.—*Glucose-6-phosphatase and Fructose-1-6-diphosphatase Activity in Liver and Tumour Tissue of Swiss Mice Fed with Thioacetamide*

| Age (months) | Tissue | Glucose-6-phosphatase Control | Glucose-6-phosphatase Treated | Fructose-1-6-diphosphatase Control | Fructose-1-6-diphosphatase Treated |
|---|---|---|---|---|---|
| 4 | Liver | 34·4±1·1 | 20·4±1·4* | 11·3±1·6 | 8·8±0·3* |
| 6 | Liver | 29·2±2·6 | 17·9±1·2* | 10·3±0·6 | 7·8±0·5* |
| 9 | Liver | 32·3±1·5 | 23·9±2·23* | 12·8±0·9 | 5·7±0·4* |
| 13 | Liver | 21·4±1·8 | 8·4±1·2* | 8·4±0·8 | 4·1±0·4* |
| 17 | Host-liver | 22·89±0·7 | 11·73±1·11* | 10·5±0·6 | 5·6±0·5* |
| | Tumour | — | 13·9±1·13* | — | 5·4±0·5* |

Values represent mean of six readings.
* Denotes statistically significant when compared with corresponding control group and $P$ value is $<0·05$.
Enzyme activities are expressed in terms of $\mu$g. of phosphorus liberated per hour mg. tissue.

Table II summarizes the activities of glucose-6-phosphatase and fructose-1-6-diphosphatase in the liver tissue of control and thioacetamide treated mice. It is evident from the table that the activities of both the enzymes in thioacetamide fed mice decreased from the age of 4 months. The extent of decrease in enzyme activities in the host liver and tumour was comparable. Moraru, Cotutiu and Streja (1967) have reported a decrease in gluconeogenesis of short term feeding of thioacetamide.

From Table III it is quite obvious that the thioacetamide treatment caused an increase in the activity of aldolase in treated mice from the age of 9 months. In

TABLE III.—*Aldolase Activity in Liver and Tumour Tissue of Swiss Mice Fed with Thioacetamide*

| Age (months) | Tissue | Control | Treated |
|---|---|---|---|
| 6 | Liver | 0·95±0·03 | 0·81±0·085 |
| 9 | Liver | 0·9±0·06 | 1·3±0·10* |
| 13 | Liver | 0·65±0·05 | 1·88±0·05* |
| 17 | Host-liver | 0·73±0·1 | 1·66±0·083* |
| | Tumour | — | 1·85±0·14* |

Values represent mean of six readings.
* Denotes statistically significant when compared with corresponding control group and $P$ value is $<0·05$.
Enzyme activity is expressed in terms of $\mu$g. of triose-phosphatase liberated per hour per mg. tissue tumour bearing mice the host liver and the tumour tissue had significantly higher enzyme activity than the corresponding control liver tissue.

TABLE IV.—*Ornithine Transcarbamylase Activity in Liver and Tumour Tissue of Thioacetamide Fed Mice*

| Age (months) | Tissue | Aspartic transcarbamylase Control | Aspartic transcarbamylase Treated | Ornithine transcarbamylase Control | Ornithine transcarbamylase Treated |
| --- | --- | --- | --- | --- | --- |
| 6 | Liver | 5·0±0·8 | 6·9±0·3 | 6·9±0·3 | 4·5±0·11 |
| 9 | Liver | 5·2±0·6 | 8·3±0·7* | 7·9±0·7 | 5·6±0·4 |
| 13 | Liver | 5·6±0·4 | 9·0±0·4* | 6·1±0·3 | 3·1±0·2* |
| 17 | Host-liver | 6·6±0·1 | 8·5±0·1* | 7·0±0·4 | 3·4±0·3* |
| | Tumour | — | 11·1±0·3* | — | 4·04±0·65* |

Values represent mean of six readings.
* Denotes statistical significance when compared with control group, and $P$ values is $<0 \cdot 05$.
Aspartic transcarbamylase activity is expressed in terms of $\mu$g. of ureidosuccinic acid formed per hour per mg. tissue.
Ornithine transcarbamylase activity is expressed in terms of $\mu$g. of Citrulline formed per hour per mg. tissue.

Table IV shows the activities of aspartic and ornithine transcarbamylase in liver and tumour tissue. It is evident from the table that aspartic transcarbamylase activity increased from the age of 9 months, whereas ornithine transcarbamylase activity decreased from the age of 13 months. Maximum change in the enzyme activities was observed in the tumour. Host liver of the tumour bearing mice had an enzyme activity comparable with that of the tumour.

Increase in aspartic transcarbamylase activity in regenerating liver and hepatomas was reported previously (Calva, Lowenstein and Cohen, 1959; Sapre, Gothoskar and Bhide, 1969*a*), as was a decrease in ornithine transcarbamylase activity in liver tissue of rats and mice fed with 3,2′dimethoxyaminoazobenzene (Bhide, Kanekar and Ambaye, 1967).

TABLE V.—*Arginase Activity in Liver and Tumour Tissue of Thioacetamide Fed Mice*

| Age (months) | Tissue | Xanthine oxidase Control | Xanthine oxidase Treated | Arginase Control | Arginase Treated |
| --- | --- | --- | --- | --- | --- |
| 6 | Liver | 1·1±0·1 | 1·1±0·2 | 1136±20 | 1225±35 |
| 9 | Liver | 1·3±0·1 | 0·7±0·08* | 1009±25 | 751±31* |
| 13 | Liver | 1·2±0·2 | 0·6±0·1* | 1189±52 | 677±42* |
| 17 | Host-liver | 1·5±0·1 | 0·5±1·2* | 1185±32 | 753±43* |
| | Tumour | — | 0·4±0·2* | — | 745±36* |

Values represent mean of six readings.
* Denotes statistical significance when compared with control group and $P$ value is $<0 \cdot 05$.
Xanthine oxidase activity is expressed in terms of $\mu$g. of xanthine used per hour per mg. tissue.
Arginase activity is expressed in terms of $\mu$g. of urea formed per hour per mg. tissue.

Table V shows the activities of xanthine oxidase and arginase in thioacetamide treated and control mice. Xanthine oxidase and arginase activity decreased from the age of 9 months. Host-liver and tumour had comparable enzyme activities.

### DISCUSSION

From the foregoing results it is apparent that thioacetamide feeding causes significant alterations in the various parameters studied in the present experiments.

Thioacetamide feeding caused a considerable loss in glycogen content and the activities of gluconeogenic enzymes from the age of 4 months. Decrease in glycogen content on feeding of carcinogenic compounds has been reported by several authors (Chang, Spain and Griffin, 1958; Orr, Price and Strickland, 1948). It may be mentioned here that it has been reported from this laboratory that even a single i.p. injection of thioacetamide (50 mg./g. body weight), selectively decreases glycogen content of the liver tissue (Sapre, Gothoskar and Bhide, 1969*b*). Hence it seems probable that continuous administration of thioacetamide should affect glycogen levels in the liver tissue of the treated mice, as early as at the age of 4 months. Furthermore it is interesting to observe that the increase in lactic acid content and the activity of aldolase appears a little later, *i.e.* at the age of 9 months. Increase in lactic acid content denotes increase in glycolytic activity and the increase in aldolase activity further supports this contention. Hence it seems that continued significant decrease in glycogen content in liver tissue of thioacetamide treated mice in later age periods, may be due to stimulated glycolytic activity of the liver tissue.

With reference to the enzymes in nucleic acid metabolism, it is interesting to note that concurrent with the increase in aspartic transcarbamylase activity, xanthine oxidase activity decreases. Sheth, Bhide and Ranadive (1968) have observed that in spontaneous mammary carcinogenesis xanthine oxidase activity in mammary tissue decreases progressively and is absent in mammary tumour. In the present experiments we have not observed total disappearance of xanthine oxidase activity in hepatomas and hence it appears that total deletion of xanthine oxidase activity is not a necessary attribute of malignancy. With reference to enzymes of the urea cycle the present work supports the observations of Burke (1962) and McLean, Reid and Gurney (1964). These authors have observed a decrease in production of urea nitrogen and in the activity of urea cycle enzymes in the livers of rats fed a carcinogenic diet.

The most interesting observation that emerges from the present experiment is that the decrease in glycogen level and activities of gluconeogenic enzymes begin to appear as early as the age of 4 months, *i.e.* 2 months after starting thioacetamide feeding. At this stage of treatment, there were no visible changes in the morphology and histology of the liver (unpublished data). On the other hand alterations in other parameters begin to appear much later, *i.e.* from the age of 9 months onwards when the hepatic cells begin to show hypertrophy and even regenerating nodules in certain areas. It is, therefore, remarkable that these significant changes in the carbohydrate metabolism appear even before the appearance of preneoplastic histological changes in the liver tissue. It now seems worthwhile to explore if such early changes in the carbohydrate metabolism of liver tissue affect the blood biochemistry as well, which may help in the early detection of possible neoplastic changes in the target organ.

The author wishes to thank Dr. (Mrs.) K. J. Ranadive, Chief, Biology Division for her constant encouragement in this project.

### REFERENCES

ARCHIBALD, R. M.—(1944) *J. biol. Chem.*, **156**, 121.—(1945) *J. biol. Chem.*, **157**, 507.
BARKER, S. B. AND SUMMERSON, W. H.—(1941) *J. biol. Chem.*, **138**, 535.
BHIDE, S. V., KANEKAR, M. G. AND AMBAYE, R. Y.—(1967) *Indian J. Cancer*, **4**, 333.

Brown, G. W. and Cohen, P. P.—(1959) *Cancer Res.*, **19**, 101.
Burke, W. T.—(1962) *Cancer Res.*, **22**, 10.
Burnett, G. H. and Cohen, P. P.—(1957) *J. biol. Chem.*, **229**, 337.
Calva, E., Lowenstein, J. M. and Cohen, P. P.—(1959) *Cancer Res.*, **19**, 101.
Chang, J. P., Spain, J. D. and Griffin, A. C.—(1958) *Cancer Res.*, **18**, 670.
Cori, G. T. and Cori, C. F.—(1952) *J. biol. Chem.*, **199**, 661.
Fiske, C. H. and Subbarow, Y. J.—(1925) *J. biol. Chem.*, **66**, 375.
Gupta, D. N.—(1956) *J. Path. Bact.*, **72**, 183.
Kim, S. and Cohen, P. P.—(1965) *Archs Biochem.*, **108**, 421.
Koritz, S. B. and Cohen, P. P.—(1954) *J. biol. Chem.*, **209**, 145.
Litwack, G., Bothwell, J. W. Williams, J. W. and Elvehjem, C. A.—(1953) *J. biol. Chem.*, **200**, 303.
McLean, P. L., Reid, E. and Gurney, M. W.—(1964) *Biochem. J.*, **91**, 464.
Moraru, I., Cotutiu, C. and Streja, D.—(1967) *Chem. Abstr.*, **66**, 36248.
Nelson, N.—(1944) *J. biol. Chem.*, **153**, 375.
Orr, J. W., Price, D. E. and Strickland, L. H.—(1948) *J. Path. Bact.*, **60**, 573.
Pogell, B. M. and McGilvery, R. W.—(1954) *J. biol. Chem.*, **208**, 149.
Ranadive, K. J.—(1957) *Coll. Pap. Lab. Anim. Bur.*, **5**, 66.
Sapre, N. N., Gothoskar, S. V. and Bhide, S. V.—(1969*a*) *Indian J. Cancer*, **6**, 219.—(1969*b*) *Indian J. exp. Biol.*, **7**, 4.
Sheth, N. A., Bhide, S. V. and Ranadive, K. J.—(1968) *Br. J. Cancer*, **22**, 833.
Sibley, J. A. and Lehninger, A. L.—(1949) *J. biol. Chem.*, **177**, 859.